# A comparison of discrete versus continuous environment in a variance components-based linkage analysis of the COGA data

**DOI:** 10.1186/1471-2156-6-S1-S57

**Published:** 2005-12-30

**Authors:** Kevin R Viel, Diane M Warren, Alfonso Buil, Thomas D Dyer, Tom E Howard, Laura Almasy

**Affiliations:** 1 Department of Epidemiology, Emory University, Atlanta, Georgia, USA; 2 Department of Pathology, Emory University, Atlanta, Georgia, USA; 3 Department of Genetics, Southwest Foundation for Biomedical Research, San Antonio, Texas, USA

## Abstract

**Background:**

The information content of a continuous variable exceeds that of its categorical counterpart. The parameterization of a model may diminish the benefit of using a continuous variable. We explored the use of continuous versus discrete environment in variance components based analyses examining gene × environment interaction in the electrophysiological phenotypes from the Collaborative Study on the Genetics of Alcoholism.

**Results:**

The parameterization using the continuous environment produced a greater number of significant gene × environment interactions and lower AICs (Akaike's information criterion). In these cases, the genetic variance increased with increasing cigarette pack-years, the continuous environment of interest. This did not, however, result in enhanced LOD scores when linkage analyses incorporated the gene × continuous environment interaction.

**Conclusion:**

Alternative parameterizations may better represent the functional relationship between the continuous environment and the genetic variance.

## Background

Generally, there is more information when a risk factor is represented by a continuous variable than a categorical variable. The resulting gain of analytical power justifies the increased effort required to collect and use data in more refined detail, i.e., packs of cigarettes per day versus smoking status. One exception to this may be the unusual circumstance in which levels of exposure below the lowest unit of measurement are sufficient to generate the outcome of interest. Another instance may be when the parameterization or constraints of a data analytical tool offer no benefit from the use of a continuous variable. The VARCOMP procedure in SAS is an example of the latter case because it allows only class variables, i.e., variables that are not continuous [1].

In this article, we examined the 12 continuous traits concerning event-related potentials (ERPs) and the continuous resting potential in the Genetic Analysis Workshop 14 (GAW14) dataset with regard to gene × environment (G × E) interaction, with the environmental exposure of interest being cigarette smoking. We considered the dichotomous indicator of habitual smoking (SMOKER), the continuous cigarette pack-years (CIGSPKY), and smoking status as a dichotomization of cigarette pack-years.

We used a variance components model with one parameterization that allowed for separate discrete environment-specific genetic and environmental standard deviations and a second parameterization that modeled the genetic standard deviation as a function of the continuous environment. The first aim of these analyses was to determine whether there is G × E interaction. The second aim was, given G × E interaction, to determine whether incorporation of the dichotomous or the continuous variable affected our ability to detect linkage in variance components based linkage analyses. Finally, given linkage, we examined whether this incorporation provided additional information about the underlying quantitative trait loci (QTL).

## Methods

### Data

We obtained data from the Collaborative Study on the Genetics of Alcoholism (COGA) provided for the GAW14. Begleiter et al. have previously described the recruitment of the study participants [2]. Bierut et al. have previously reported the study design and defined the phenotypes of interest [3]. These data contain 13 electrophysiological phenotypes: TTTH1-TTTH4, TTDT1-TTDT4, NTTH1-NTTH4, and ECB21. These phenotypes are ERPs, i.e., neuroelectric activity generated in response to stimulus, with the exception of ECB21, which is the spontaneous electrical activity of the brain of a relaxed subject. Electrodes attached to the scalp of the subject record the activity transmitted through a conductive gel. Spatial and temporal characteristics differentiate the various ERPs. The data also include the age of the individual at collection of the ERP data (ERPAGE), which may have occurred after the initial recruitment. A dichotomous variable, SMOKER, indicates habitual smoking, defined as smoking a pack or more of cigarettes a day for a period of at least six months. A related continuous variable, CIGPKYRS, is the number of packs of cigarette smoked per day for one year. We created indicator of any smoking (SMK_STATUS) by dichotomizing CIGPKYRS into a group with zero consumption and another with any consumption.

### Model parameterization

We parameterized a gene × discrete environment (G × discrete E) variance components model to allow for separate environmental-specific genetic and environmental SD. Table 1 specifies the general form of the three possible covariance matrices for this parameterization. This model allowed one genetic SD for smokers and another genetic SD for nonsmokers. Specifically, we tested whether the genetic SDs were the same in smokers (σ_xg_) and nonsmokers (σ_yg_) and whether the genetic correlation (ρ_g_) between smokers and nonsmokers differed from 1. When the genes in both smokers and nonsmokers that influence the trait comprise identical sets, ρ_g _= 1, whereas when the genes in smokers and nonsmokers that influence the trait comprise completely different, nonoverlapping sets of genes, ρ_g _= 0. In this description, smoker is general for either SMOKER or SMK_STATUS. Towne et al. [4] describe more fully this type of variance components model for G × discrete E.

The corresponding parameterization of a gene × continuous environment (G × continuous E) model allowed the genetic SD (σ_g_) to be a linear function of cigarette pack-years. This involves two parameters, a genetic SD (σ_g_) that applies at the mean value of cigarette pack-years and a slope (β) for change in the natural logarithm of the genetic SD with cigarette pack-years. Specifically,

σ^2^_g _= exp [α + β(CIGPKYRS - μ_CIGPKYRS_)]   (1)

ρ_g _= exp [-λ|CIGPKYRS_i _- CIGPKYRS_j_|]   (2)

Under this parameterization, the natural logarithm of the genetic correlation (ρ_g_) decreases linearly with increasing disparity in CIGPKYRS, such that individuals with the same CIGPKYRS have ρ_g _= 1 and individuals with increasing differences in CIGPKYRS have decreasing ln(ρ_g_) with slope -λ. Almasy et al. [5] further described this G × continuous E model. We tested whether the β was different from zero by employing a likelihood ratio test with one degree of freedom for significance testing. Two models, which differed only in that one was subject to the constraint β = 0, generated the likelihoods for this test.

### Linkage analysis

We performed whole-genome linkage analyses that incorporated a G × E interaction and linkage analyses that did not incorporate a G × E interaction. For all of the analyses, we used SOLAR [6]. For the linkage analyses we used the microsatellite-based genotypes. The measured covariates included ERPAGE, sex, the square of ERPAGE, the interaction of sex with both ERPAGE and the square of ERPAGE, and, when incorporating G × E interactions, smoking status (SMOKER).

### Akaike's Information Criterion (AIC)

For the various models, we calculated AIC [7] and scaled the trait values by multiplying them by 10 for ease of computation.

## Results

### G × discrete E

We found evidence of a genotype-by-smoking interaction only for TTTH1, using either SMOKER or SMK_STATUS as the discrete environment. Table 2 shows the genetic SD specific to the smoking (x) and to the nonsmoking environment (y). Additionally, Table 2 presents the AIC for the unconstrained model, the model subject to the constraint σ_xg _= σ_yg_, and the model subject to the constraint ρ_g _= 1. Though the differences in AIC between the models were unimpressive, the models subject to the constraint ρ_g _= 1 consistently had the lowest AIC, except for the trait NTTH3, in which it equaled that for the model subject to the constraint σ_xg _= σ_yg_. For all outcomes, including that of TTTH1, there was no difference in the source of genetic effects between the habitual smokers and non-habitual smokers, i.e., the genetic correlation (ρ_g_) was not statistically different from 1. When SMK_STATUS was the discrete environment, however, there was evidence that ρ_g _for TTTH3 and for TTTH4 differed statistically from 1 (*p *= 0.022 and *p *= 0.025, respectively), i.e., the sets of genes in smokers and nonsmokers that influence the trait were not identical.

Upon performing a linkage analysis without incorporating the G × discrete E interaction we found a maximum LOD score of 3.4116 at chromosome 7, 157–158 cM. Upon incorporating the G × discrete E interaction, for which SMOKER was the discrete environment of interest, we found a maximum LOD of 3.6190 at the same location. Table 3 contrasts the LOD scores found in the analyses that incorporated the genotype × smoking interaction versus those that did not.

The genetic SD due to the locus at chromosome 7, 157–158 cM among smokers was not significantly different from that of nonsmokers, σ_qx _= 0.338 and σ_qy _= 0.335, respectively. The difference in residual polygenic effect among smokers and nonsmokers, σ_gx _= 0.271 and σ_gy _= 0.550, respectively, appears intriguing, but remains statistically insignificant (*p *= 0.36). For the locus at chromosome 6, 96 cM, there is a statistical difference *(p *= 0.0139) between the genetic variation due to the locus among smokers and that among nonsmokers.

### G × continuous E

We found evidence of G × continuous E interaction for NTTH1, NTTH4, and TTTH4, but not for TTTH1. In each case, β was positive, indicating that the genetic variance increased with increasing cigarette pack-years. Table 4 presents the results of these analyses and the AIC for the unconstrained and constrained models (β = 0). Given that the likelihood ratio test tested the constraint, it is consistent that the cases in which β was significantly different from zero resulted in a lower AIC for the unconstrained model. The linkage analyses with the G × continuous E interaction did not improve the LOD scores. The AIC from the models with the continuous parameterizations were lower than those from the corresponding models with the discrete parameterizations.

## Conclusion

These analyses suggest that the parameterization using the continuous environment seems to be a better choice as more results of G × E investigations were significant for the continuous environment and the resulting AIC were lower. Whether this parameterization conveys greater power, however, is unknown. Further, as indicated by the linkage analyses, implementation of this parameterization may be sensitive to the particular functional relationship of the environment to the genetic variance. In particular, alternative parameterizations, such as described by Diego et al. [8], may provide directions for further exploration.

## Abbreviations

AIC: Akaike's information criterion

COGA: Collaborative Study on the Genetics of Alcoholism

GAW14: Genetics Analysis Workshop 14

ERP: Event-related potentials

G × E: Gene × environment

QTL: Quantitative trait loci

## Authors' contributions

KRV performed statistical analyses and wrote the manuscript. DMW performed the linkage analyses that did not incorporate interaction and assisted in editing the manuscript. AB provided programming assistance and statistical analyses. TDD provided the IBD matrices for the linkage analyses. THE assisted with interpretation of results. LA conceived the study and provided direction, in addition to editing the manuscript. All authors read and approved the final manuscript.

## Figures and Tables

**Table 1:** The general form of covariance matrices for the G × discrete E parameterization

Subject i smokes	Subject j smokes	Covariance Matrix
1	1	**Ω **= 2 **Φ **σ^2^_xg _+ **I **σ^2^_xe_
0	0	**Ω **= 2 **Φ **σ^2^_yg _+ **I **σ^2^_ye_
1	0	**Ω **= 2 **Φ **σ_xg _σ_yg _ρ_g _+ **I **σ_xe _σ_ye_

**Table 2:** Genetic standard deviations specific to the discrete environment, the genetic correlations, and the corresponding AIC^a^

	Model			AIC^a^		
		
Trait	σ_xg_^b^	σ_yg_^b^	ρ_g_^c^	Unconstrained	σ_xg _= σ_yg_	ρ_g _= 1
CB21	3.8850	3.5510	1.0000	3700.2200	3698.5900	3698.2200
NTTH1	0.2400	0.2080	1.0000	3005.3000	3003.5600	3003.3000
NTTH2	0.4380	0.4080	1.0000	3521.6300	3519.7700	3519.6300
NTTH3	0.5080	0.5010	1.0000	3563.2000	3561.2000	3561.2000
NTTH4	0.4050	0.3470	1.0000	3552.6800	3551.1200	3550.6800
TTDT1	0.3950	0.5060	1.0000	3795.3100	3794.0500	3793.3100
TTDT2	0.5870	0.8050	1.0000	4036.0500	4037.1500	4034.0500
TTDT3	0.7410	0.9570	1.0000	4302.4600	4302.6100	4300.4600
TTDT4	0.8710	1.0390	1.0000	4479.1500	4478.1700	4477.1500
TTTH1	0.463^d^	0.653^d^	0.9020	3452.8800	3455.1500	3450.9700
TTTH2	0.7130	0.7360	1.0000	4022.0400	4020.0800	4020.0400
TTTH3	0.7460	0.8060	1.0000	4059.3000	4057.6100	4057.3000
TTTH4	0.6140	0.6420	1.0000	3850.9600	3849.0500	3848.9600

^a^AIC, Akaike's Information Criteria

^b^Genetic standard deviation within smokers (x) and nonsmokers (y)

^c^Genetic correlation between smokers and nonsmokers. None were significantly different from 1, i.e., the sets of genes influencing the traits were identical between smokers and nonsmokers.

^d^*p *= 0.039

**Table 3:** Linkage analyses of TTTH1 with and without incorporation of G × discrete E interaction

		LOD without G × E	LOD with G × E	QTL SD		Residual genetic SD	
					
Chromosome	cM			Smokers	Nonsmokers	Smokers	Nonsmokers
1	212	1.97	2.63	0.32	0.33	0.31	0.56
6	96–97	1.68	2.25	0.40^a^	0.77^a^	0.28	0.27
7	157–158	3.40	3.62	0.34	0.34	0.27	0.55

^a^*p *= 0.0139

**Table 4:** The genetic SD change as a linear function of the continuous CIGPKYRS and corresponding AIC^a^.

Trait	βσ_g_	βΔσ_g_	χ^2^	*p*-value	AIC, unconstrained	AIC, constrained
ECB21	1.383	-1.900 × 10^-3^	0.221	0.638	3625.52	3623.73
**NTTH1**	**1.093**	**7.930 × 10^-3^**	**5.640**	**0.018**	**2952.57**	**2956.21**
NTTH2	1.470	4.879 × 10^-3^	2.652	0.103	3446.97	3447.63
NTTH3	1.564	4.024 × 10^-3^	3.282	0.070	3471.96	3473.24
**NTTH4**	**1.481**	**8.989 × 10^-3^**	**7.365**	**0.007**	**3496.43**	**3501.80**
TTDT1	1.425	-4.964 × 10^-3^	0.145	0.703	3729.57	3727.71
TTDT2	1.642	-2.365 × 10^-3^	0.243	0.622	3973.37	3971.61
TTDT3	1.885	-5.396 × 10^-3^	0.632	0.426	4233.12	4231.76
TTDT4	2.094	-7.761 × 10^-3^	1.264	0.261	4403.23	4402.49
TTTH1	1.643	-1.538 × 10^-3^	0.191	0.662	3388.68	3386.87
TTTH2	1.943	1.469 × 10^-3^	0.281	0.596	3956.94	3955.22
TTTH3	2.011	4.015 × 10^-3^	2.721	0.099	3993.69	3994.41
**TTTH4**	**1.874**	**8.027 × 10^-3^**	**12.234**	**4.69 × 10^-4^**	**3783.90**	**3794.13**

^a^AIC, Akaike's Information Criteria

Bold signifies that βΔσ_g _was significantly different from 0 (*p *≤ 0.05)
